# Food matrix impacts bioaccessibility and assimilation of acid whey-derived milk fat globule membrane lipids in Caco-2 cells

**DOI:** 10.3389/fnut.2023.1177152

**Published:** 2023-05-09

**Authors:** Erica Kosmerl, Victoria Martínez-Sánchez, María V. Calvo, Rafael Jiménez-Flores, Javier Fontecha, Antonio Pérez-Gálvez

**Affiliations:** ^1^Department of Food Science and Technology, The Ohio State University, Columbus, OH, United States; ^2^Group of Chemistry and Biochemistry of Pigments, Instituto de la Grasa (CSIC), Sevilla, Spain; ^3^Food Lipid Biomarkers and Health Group, Institute of Food Science Research (CSIC-UAM), Madrid, Spain

**Keywords:** milk fat globule membrane, matrix influence, lipid metabolism, *in vitro* digestion, bioaccessibility

## Abstract

The milk fat globule membrane (MFGM) imparts human health benefits ranging from improved immune system, gut, and brain function to improved cardiometabolic health. The industry’s growing interest in introducing MFGM-enriched foods requires scientific evidence that the benefits derived from this compound are not affected by the formulation or processes that may alter its function, such as the digestion process. In this study, the impact of food matrices and supplementation levels on the bioaccessibility and assimilation of MFGM lipids in cell culture was investigated. Three food matrices including a protein-rich jelly, carbohydrate-rich cookie, and a carbohydrate- and fat-rich cookie with sunflower oil (SF-cookie) were supplemented with an MFGM ingredient derived from cottage cheese acid whey at 2, 5, and 10% (w/w). Each formulation underwent simulated digestion consisting of oral, gastric, and intestinal phases, and the micellar fraction was collected for both analysis and lipid assimilation in Caco-2 intestinal cells. The micellar fractions were diluted and applied to the cells for 4 h. A lipidomic approach was used to assess the lipid profiles of micellar fractions and intestinal cells. The micelles from digested jellies, cookies, and SF-cookies containing MFGM showed a distinct separation using partial least squares discriminant analysis (PLS-DA). Both correlation loadings and variable importance in projection (VIP) scores demonstrated a tendency of MFGM polar lipids (ceramides, glucosylceramides) for micelles from digested jelly, whereas micelles from digested cookies were associated with MFGM neutral lipids (free fatty acids, cholesterol, etc.). The effect of supplementation level on the micellar lipid profiles reinforced this pattern. The lipid profiles of intestinal cells after incubation with the micellar fractions differed considerably from the corresponding micellar lipid profiles. Specifically, the SF-cookie-treated cells were associated with a greater abundance of PUFA relative to jelly- and cookie-treated cells; however, increasing MFGM supplementation showed irregular patterns and rearrangement of cellular lipid profiles, suggesting the cells’ role in regulating lipid metabolism in response to nutritional stimuli. The nature of lipid micellarization and assimilation in intestinal cells from MFGM-containing food formulations echoes the complexity of lipids inherent to the MFGM itself, suggesting the need for application-based MFGM supplementation.

## 1. Introduction

The milk fat globule membrane (MFGM) has attracted significant attention for its ability to convey health benefits across all ages. The role of the MFGM in neurocognition, immune and gut function, and cardiometabolic health has been an increasingly relevant topic of nutritional studies. Clinical trials and *in vivo* experiments have revealed the role of MFGM in supporting brain health and cognitive function across the lifespan by improving memory and learning capacity in both infants (1) and aging animal models (2). Dietary MFGM also shapes the gut microbiota and strengthens gut barrier function via changes in tight junction protein expression, villi height and crypt depth, and goblet cell number (3). Regarding immune function, the MFGM mitigates endotoxin-induced systemic inflammation (4) and modulates high fat diet-induced inflammatory markers (5). Others studies have also reported the benefits of MFGM in the dose-dependent regulation of dyslipidemia and markers of cardiometabolic diseases through alterations in serum cholesterol (6). Ultimately, these diverse health benefits, positions the MFGM as an ideal ingredient to supplement various food matrices.

The MFGM has a unique composition in complex lipids and proteins (around 40 and 60%, respectively) that originate from the mammalian secretory cells during milk secretion (7). Among its major lipid constituents are phospholipids (i.e., phosphatidylethanolamine (PE), phosphatidylcholine (PC), phosphatidylserine (PS), phosphatidylinositol (PI)), and sphingolipids (e.g., sphingomyelin (SM)), glycosphingolipids (i.e., gangliosides, ceramides), cholesterol (CHOL), free fatty acids (FFA), and other minor lipids, which are organized into a very complex characteristic trilayer structure upon milk secretion (8). In line with the current trends of valorizing waste and byproducts of the dairy industry, MFGM-enriched ingredients have been isolated from sweet whey cream from cheese making, buttermilk from butter churning, and butter serum from butter oil production (9, 10), and more recently, acid whey (AW) derived from cottage cheese and Greek yogurt manufacture (11, 12). As Greek yogurt and cottage cheese production has increased, the dairy industry must address AW, which poses significant disposal, upcycling, and valorization challenges due to its high mineral content and low pH. MFGM-enriched ingredients from AW are obtainable through the application of a series of precipitation, centrifugation, and ultrafiltration steps with very limited thermal treatment (13). The valorization of AW into MFGM-enriched ingredients offers two concomitant solutions: (i) a reduction in waste disposal and (ii) an innovative source of bioactive lipids to benefit health.

The application of MFGM ingredients, such as AW-derived MFGM, into food products requires a foundational understanding of the relationship between a food product and digestion, absorption, and metabolism to adequately deliver their desirable health benefits. Lipid digestion and absorption is a complicated process that involves (i) the liberation of bioactive lipids from a food matrix, (ii) formation of micelles for solubilization, (iii) uptake by the intestinal epithelium, (iv) chylomicron packaging, and (v) release into the circulation via the lymph (14). Lipid bioaccessibility is referred to as the proportion of lipids liberated from a food matrix and subsequently rearranged into micelles that are available for uptake by enterocytes. In essence, bioaccessibility is a prerequisite for bioactive lipophilic molecules to benefit the host. Dietary factors, such as food matrix structure, food composition, and supplementation level (15), all contribute to the bioaccessibility of lipids, along with physiological factors, such as gut microbiota, genetics, and age (14).

In the present study, we focused on two key factors which can potentially impact the bioaccessibility of MFGM lipids. Our first aim was to determine the influence of food matrix composition and supplementation level on the bioaccessibility of MFGM lipids using 3 distinctive food matrices (protein-rich, carbohydrate-rich, or carbohydrate- and fat-rich matrices). Our second objective was to assess the assimilation of AW-MFGM lipids after micellarization into enterocytes using Caco-2 cell culture and lipidomic analysis.

## 2. Materials and methods

### 2.1. Isolation and production of MFGM ingredient from acid whey

An MFGM ingredient derived from acid whey (AW-MFGM) was produced in The Ohio State University Dairy Pilot Plant capable of processing up to 500 gallons of acid whey as described in a previously patented process (13). Fluid acid whey from cottage cheese manufacturing was generously donated by Superior Dairy (Wooster, OH, United States). The acid whey was first filtered using cheese cloth to remove residual curds and large sediment. Ultrafiltration and diafiltration (4X) with a 50 kDa molecular weight cut-off (MWCO) spiral wound membrane was used to remove minerals and concentrate the product 50% by volume using a Model R Pilot Scale Membrane Filtration Unit (GEA, Düsseldorf, Germany). The concentrated retentate, containing caseins, whey proteins, phospholipids, and residual fat, was separated using a pilot scale centrifugal cream separator (SPX, Charlotte, NC, United States). The resulting ‘sludge’ or precipitate that remained in the bowl of the separator was resuspended in 100 mL of DI water per 500 g of precipitate and treated with 30 mM tri-sodium citrate (600 mL per 1 L of resuspended precipitate) at 4°C for 20 min to precipitate protein aggregates. After subsequent separation using a cream separator, the supernatant was treated with NH_4_OH to reach a final pH of 11.0 for 20 min at 4°C. Ultrafiltration and diafiltration (4X) with a 50 kDa MWCO membrane was then used to remove solubilized caseins and residual citrate and ammonium hydroxide. The retentate (containing MFGM phospholipids and residual fat) was then spray-dried using a pilot scale spray-drying system (CIT-PSD-P10, Col-Int Tech, Irmo, SC, United States). The complete lipid composition of the AW-MFGM ingredient is described in Supplementary Table S1.

### 2.2. Food formulations and experimental design

Three different food formulations were prepared to study the different effects of matrix composition on the bioaccessibility of AW-MFGM: protein-rich food matrix (jelly), carbohydrate-rich food matrix (cookie), and lipid- and carbohydrate-rich food matrix (cookie with sunflower oil (SF-cookie)). Prior to preparation, AW-MFGM was hydrated for 18 h at 4°C on a rocking platform (VWR Rocking platform, 95 rpm) to enhance dissolution. After hydration, AW-MFGM was incorporated into each food matrix at three different concentrations: 2, 5, and 10% (w/w). The amount for each concentration was added and dissolved directly in each matrix. For the protein-rich matrix, 35 g of sugar, 3 g of jelly, the necessary weight of AW-MFGM to reach the supplementation concentrations, and 62 g of water were mixed and heated for complete dissolution. The mixture was then cooled at 4°C until complete solidification of the matrix. For the carbohydrate-rich matrix, 140 g of egg white, 100 g of white sugar, 150 g of all-purpose flour and the respective amounts of MFGM were mixed and baked for 15 min at 180°C. For the lipid- and carbohydrate-rich matrix, 100 g of egg white, 80 g of sunflower oil, 100 g of white sugar, 150 g of all-purpose flour and the respective amounts of MFGM were mixed and baked using the same conditions as described previously. Each food preparation was kept frozen (−20°C) until needed.

The experimental design for the study was as follows: three independent *in vitro* digestion procedures were performed with three replicates of each sample. Each sample is defined as the different food matrices with different AW-MFGM concentrations described above: jelly with 0% (control), 2, 5 and 10% MFGM supplementation; cookie with 0% (control) 2, 5, and 10% MFGM supplementation; and SF-cookie with 0% (control), 2, 5, and 10% MFGM supplementation. The static INFOGEST *in vitro* digestion protocol (16) with modifications described by Viera et al. (17) aimed to isolate micellar fractions, that, once collected, were filtered and divided to perform the extraction of the lipid present in the micelles and the cell experiments to determine the bioaccessibility of MFGM lipids. Cells were collected and the lipid fraction was extracted. Lipids from micellar fraction and cells were used for further analysis and characterization.

### 2.3. *In vitro* digestion

The recommendations and conditions described for *in vitro* digestion were applied from the static INFOGEST digestion protocol (16), which follows the common phases of human digestion (oral, gastric, and intestinal phases). Modifications described by Viera et al. (17) were applied to isolate the micellar fraction containing digested lipids. The simulated digestion fluids were prepared in advance and frozen (−20°C) until needed. The day of the experiment, the solutions were thawed and both CaCl_2_ and the required enzymes were prepared immediately before use. Due to the pH requirement of each digestion phase, preliminary experiments with each food matrix and each supplementation level of MFGM were performed to pre-determine the volume of 5 M HCl and 5 M NaOH necessary to adjust final pH for the gastric and intestinal phases. Three replicates of each sample in three different *in vitro* digestion procedures were performed.

For the oral phase, an initial weight of 2.5 g of each food matrix supplemented with MFGM was mixed with ultrapure water to obtain a total initial weight of 5 g and simulation of mastication process was performed. To obtain a final volume of 10 mL, each sample was mixed with 4 mL of warm simulated salivary fluid (SSF) electrolyte stock solution, 25 μL of 0.3 M CaCl_2_ × (H_2_O)_2_, 750 μL of α-amylase (1,000 U/mL), and ultrapure water and incubated for 2 min at 37°C on a rocking platform (VWR Rocking platform, 95 rpm). For the gastric phase, 8 mL of warmed simulated gastric fluid (SGF) electrolyte stock solution, 5 μL of 0.3 M CaCl_2_ × (H_2_O)_2_ and the necessary volume of 5 M HCl to adjust the pH to 3.0 were added to the oral bolus, following with the addition of 667 μL of pepsin (60,000 U/mL) and ultrapure water to adjust the volume to 20 mL. The gastric phase was run for 2 h with the same conditions as described before (37°C at 95 rpm on a rocking platform). For the intestinal phase, 8 mL of warmed simulated intestinal fluid (SIF) electrolyte stock solution, 3 mL of bile salts solution (10 mM) and the necessary volume of 5 M NaOH to adjust the pH to 7.0 were added to each sample and incubation was continued for 30 min under same conditions as the gastric phase. Afterwards, 40 μL of 0.3 M CaCl_2_ × (H_2_O)_2_, 5 mL of trypsin (100 U/mL), and ultrapure water were added to a final volume of 40 mL. The incubation conditions for the intestinal phase (2 h) remained constant from the previously described conditions in the gastric phase. To obtain the lipid micelles formed during the digestion, the samples were transferred to centrifuge tubes and centrifuged for 45 min at 4°C (15,500 × g). Then, 20-mL of the micellar fraction (MF) was collected from the middle part of the centrifuged tube using a syringe, filtered through a 0.45 μm nylon filter, and divided in 10 mL for subsequent lipid extraction and 10 mL for following cell experiments. Samples were stored at −20°C until subsequent analysis.

### 2.4. Caco-2 cell culture

Caco-2 cells were routinely cultured in 75 cm^2^ flasks in complete medium containing low glucose DMEM medium with L-glutamine and sodium pyruvate (Gibco) and supplemented with 10% heat-inactivated fetal bovine serum (FBS, Thermo Fisher), 1% non-essential amino acids (Gibco), and 1% penicillin/streptomycin (Gibco). Cells were maintained under a 5% CO_2_ atmosphere at 37°C. Weekly subculture was performed when the cell culture reached 70–90% confluency using trypsin–EDTA (0.25%, Gibco). For these experiments, cells were seeded on 96-well plates (Corning) and 6-well plates (Corning) at a density of 2.1 × 10^4^ per cm^2^ and grown for 21 days changing the media every other day, ensuring the complete differentiation of the cell culture. Twenty-four hours before the day of the experiment, complete medium of the 6-well plates was replaced to serum-free medium. The day of the experiment, filtered MF replicates from the same concentration and same digestion were thawed and pooled together, obtaining three digestion replicates of each food matrix and supplementation. Each digestion replicate was diluted accordingly to avoid cell toxicity (previously tested): for jelly and cookie, the MF was diluted 1:2 in Hank’s balanced salt solution (HBSS, Gibco) and then to 1:3 in serum-free medium, while for SF-cookie a dilution of 1:4 with HBSS and subsequent dilution of 1:3 in serum-free medium was used. After the preparation of the samples, Caco-2 cells were incubated with the MF for 4 h at 37°C and 5% CO_2_ atmosphere. All dilutions were pre-warmed before use. Each experiment contained 3 replicates of control cells with only medium and 3 replicates of 0, 2, 5, and 10% MFGM supplementation with the respective food matrix. After the incubation, the medium was discarded, and cells were washed twice with 0.5 mL of ice-cold 10 mM sodium taurocholate in PBS and the cell monolayer was scraped with 1 mL of 10% ethanol in PBS four times. Aliquots of 50 μL were taken for protein concentration determination by BCA protein assay (Novagen) and the rest were kept frozen at −20°C.

#### 2.4.1. BCA protein quantification

To normalize the data obtained after the cell experiment by protein concentration, BCA protein assay was performed following the manufacturer’s instructions. Briefly, 25 μL of each sample and bovine serum albumin (BSA) standard were loaded on a 96-well plate (Corning) and mixed with 200 μL of BCA working reagent. After shaking for 30 s, the plate was incubated for 30 min at 37°C and cooled to room temperature before reading the absorbance at 570 nm. Protein concentrations were calculated using the standard curve.

#### 2.4.2. Neutral red assay for cell viability

To determine cell viability after incubation with the different MF replicates in 96-well plates, a neutral red uptake assay was performed (18). After MF treatment, cells were washed with HBSS and 50 μL of complete medium containing neutral red (50 μg/mL) was added. Plates were incubated for 30 min at 37°C and 5% CO_2_ atmosphere. The staining solution was then discarded, and the cells were washed with HBSS twice. Neutral red was extracted from the viable cells using 75 μL of 1% acetic acid in 50% ethanol (v/v). The absorbance was measured at 570 nm using a plate reader.

### 2.5. Isolation of lipids from food formulations, micellar fractions, and cell culture

To extract the lipid fraction present in the food matrices, MF, and Caco-2 cell culture, the simplified method by Löfgren et al. (19) with the modifications made by Calvo et al. (9) was followed. For liquid samples (MF and cell culture), the volume ratios between sample and solvent were as follows: the liquid sample was mixed with methanol in 1:1 volume ratio and vortexed for 10 min at room temperature. Then, 2 volumes of dichloromethane were added, followed by vortexing for 10 min, 1 min of ultrasonication (JP Selecta, Barcelona, Spain), and 10 min of vortexing. Then, 0.4 volumes of 1% of acetic acid was added and vortexed again for 10 min. Afterwards, the mixture was centrifuged for 20 min at room temperature (400 × g). The bottom layer (organic phase) was extracted using a syringe and transferred to Eppendorf tubes, flushed with N_2_ gas until dry, weighed, purged with N_2_ gas, and kept frozen (−20°C) until further chromatographic analysis.

### 2.6. Lipid analyses

Separation of lipid classes was accomplished by using an HPLC system (model 1,260; Agilent Technologies Inc. Palo Alto, CA, United States) coupled with an Evaporative Light-Scattering Detector (SEDEX 85 model; Sedere SAS, Alfortville, Cedex, France) as described by Castro-Gómez et al. (20). The determination of molecular species of triacylglycerols (TAG) and CHOL by GC-FID was performed on a Clarus 400 GC (Perkin Elmer Ltd., Beaconsfield, United Kingdom) equipped with an automatic split/splitless injector and a flame ionization detector. The experimental chromatographic conditions were the same as published by Fontecha et al. (21). In regard to the fatty acid (FA) composition of the extracts, the acid–base methylation method reported by Castro-Gomez et al. (20) was used to obtain FA methyl esters (FAME), that were then analyzed by GC-FID as described by Calvo et al. (9).

### 2.7. Statistical analysis

The profile of lipid features in micelles isolated from digested food formulations and from cell cultures, were analyzed in the model including ‘Food matrix’ and ‘Supplementation level’ factors and their interaction. Lipid classes data were normalized by the median and Pareto scaled. Data quality was assessed through multivariate analysis comparing individual samples’ data against the pooled samples. Partial least squares discriminant analysis (PLS-DA) was applied to the data for multivariate testing, yielding classification models. Then, data were subjected to ANOVA analysis with raw-*p* values adjusted for multiple hypothesis testing at a false discovery rate of 5% (FDR ≤ 0.05). Interpretation of the variation induced by the factors included in the design and their combinations in the lipid classes were assessed by ANOVA and the interaction models. For comparison of the lipid profiles in micelles with those in cell cultures, normalized data of paired samples were subjected to hierarchical cluster analysis using the Ward algorithm and measuring Euclidean distance. The IBM SPSS Statistics for Windows, version 28 (IBM Corp., Armonk, N.Y., United States) was used for data analysis.

## 3. Results

### 3.1. Micellar fractions

**Figure 1:**
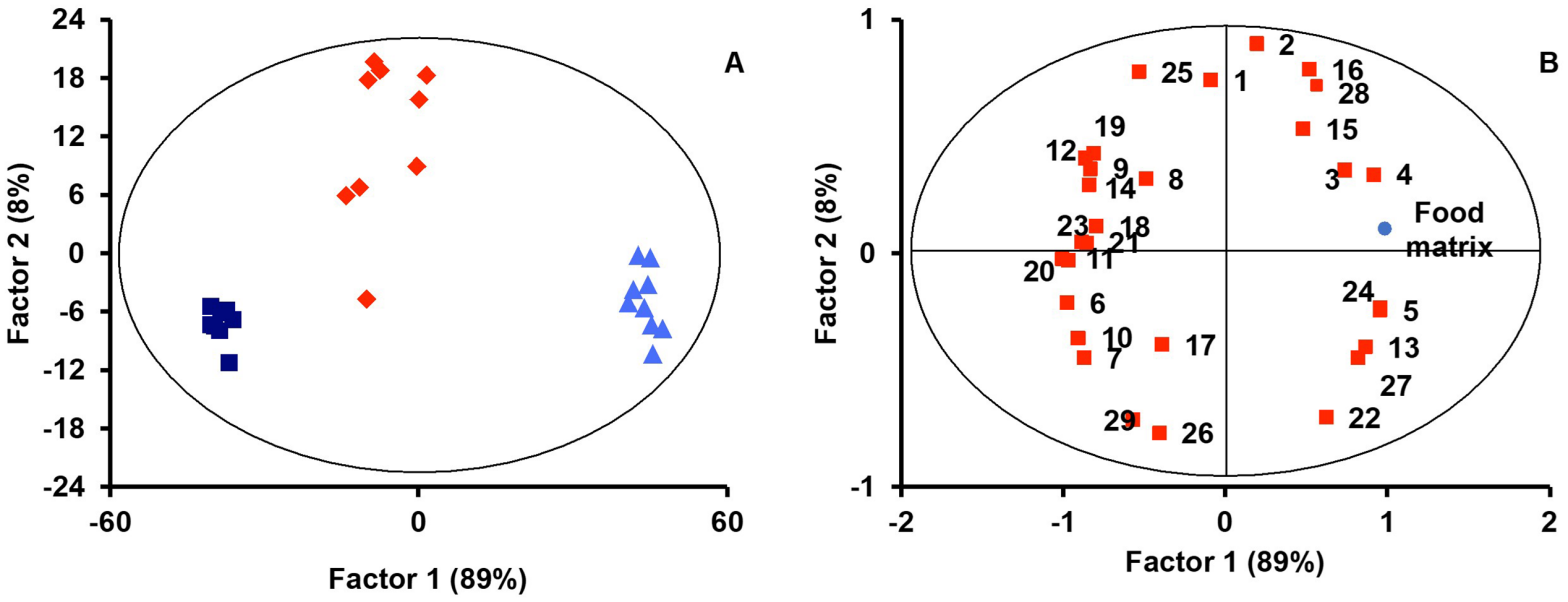
PLS-DA scores **(A)** and correlation loadings **(B)** for lipid profiles in micelles considering the ‘Food matrix’ factor (jelly, dark blue squares; cookie, red diamonds; SF-cookie, light blue triangles). Lipid features are identified with numbers as follows: 1, Cholesterol esters (CE); 2, TAG; 3, DAG; 4, FFA + CHOL; 5, MAG; 6, Glucosylceramides (GlucCer); 7, PA; 8, PC; 9, SM; 10, ƩPL; 11, Ceramides; 12, ƩNeutral lipids; 13, ƩPolar lipids; 14, CHOL; 15, C4:0; 16, C5:0; 17, C10:0; 18, C14:0; 19, C16:0; 20, C16:1 c9; 21, C18:0; 22, C18:1 c9; 23, C18:1 c11; 24, C18:2 c9 c12; 25, SFA; 26, MUFA; 27, PUFA; 28, SCFA; 29, MCFA + LCFA.

PLS-DA with lipid profiles from the digesta micellar fractions for the ‘Food matrix’ factor enabled an initial identification of the most important discriminant variables. Figure 1A shows a scatter plot of samples grouped according to the first two components with an ellipse bounding the samples within the 95% confidence interval. A clear separation was observed between lipid profiles in micelles from digested jellies, cookies, or cookies with sunflower oil (SF-cookie). Table 1 includes the values corresponding to the total explained variance (*R*^2^) and the predictable variation (*Q*^2^) of the PLS-DA model, from which Figure 1A was arranged, as well as for those paired comparisons between each food formulation made for MFGM supplementation. According to results noted in Table 1, the separation between samples in the latter comparisons was also significant. Figure 1B represents the corresponding correlation loadings for the model where the comparison between the three food matrices was made. This figure helps to detect those significant lipid features and the associations between them and the analyzed factor. Food matrices trending toward higher fat content (SF-cookie) positively correlates with a richer micellar fraction in monoacylglycerols (MAG), diacylglycerols (DAG), and FFA + CHOL, while the jelly matrix correlates well with glucosylceramides (GlucCer), total contents of ceramides, and Ʃ phospholipids (Ʃ PL). In the middle of this diverse trend, the cookie formulation had no association for a higher or lower presence of specific MFGM lipids in the micellar fraction. This observed trend is reproduced when the food formulations supplemented with MFGM were compared in pairs (see Table 1). As noted above, MFGM lipids in the micellar fractions allowed us to discriminate between the different food formulations applied for supplementation. To follow the study of those lipid features that were most relevant in their separation, we analyzed the variable importance in projection (VIP). The lipids with significant values for VIP scores (VIP > 1) are represented in Table 2. As expected, a majority presence of polar lipids in micellar fractions was associated with the jelly matrix as GlucCer, ceramides, and total polar lipid content reached significant VIP scores for jelly vs. both cookies’ matrices. Additionally, the combined FFA + CHOL fraction ranked higher among the VIP scores. On the other hand, the comparison between both types of cookies formulations pointed to neutral lipid classes characterizing the micelles. The VIP scores showed significant differences in polyunsaturated fatty acids (PUFA), monounsaturated fatty acids (MUFA), MAG, and the above-mentioned composite fraction of FFA + CHOL, among other neutral lipid classes between food matrix micelles.

**Table 1:** PLS-DA performance considering lipid profile in micelles from digested MFGM-supplemented food matrices or in cell cultures after incubation with those micellar fractions.

	Accuracy^a^	*R*^2^	*Q*^2^	Permutation test^b^
Lipids in micelles				
Jelly vs. Cookie	1.000	0.976	0.950	*p* < 0.01
Jelly vs. SF-cookie	1.000	0.992	0.972	*p* < 0.01
Cookie vs. SF-cookie	1.000	0.957	0.863	*p* < 0.01
All	0.926	0.843	0.732	*p* < 0.01
Lipids in cells				
Jelly vs. Cookie	0.667	0.325	0.01	*p* > 0.05
Jelly vs. SF-cookie	1.000	0.929	0.865	*p* < 0.05
Cookie vs. SF-cookie	0.889	0.666	0.275	*p* > 0.05
All	0.371	0.528	0.301	*p* < 0.05

a Calculated for 3 components.

b Separation distance B/W.

**Table 2:** Variable importance in projection scores (VIP) for lipid profile in micelles or in the corresponding cell cultures for each food matrix comparison pair, and for all food matrices.

	Jelly vs. Cookie		Jelly vs. SF-cookie		Cookie vs. SF-cookie	All
Lipids in micelles						
FFA + CHOL	1.551		1.762		1.838	
MAG			1.996		2.347	
GlucCer	2.238		1.012			2.407
Ceramides	3.682		1.795			3.863
PA	1.213					1.319
Σ PL	1.239					1.339
Σ Polar lipids			2.077		2.276	
C5:0	1.188					1.074
C18:1c9			1.025		1.175	
C18:2c9c12			1.803		1.931	
MUFA					1.042	
PUFA			1.580		1.705	
SCFA	1.289					1.019
MCFA + LCFA			1.655		1.878	
Lipids in cell culture						
CE	–		2.371		–	–
FFA + CHOL	–		1.221		–	–
Σ Polar lipids	–^a^		1.804		–	–
C5:0	–		1.759		–	–
C18:2 c9 c12	–		1.604		–	–
SFA	–		1.949		–	–
PUFA	–		1.373		–	–
SCFA	–		1.836		–	–

Only significant values (VIP > 1) are included. Performance of the PLS-DA models is detailed in Table 1.

a PLS-DA models were not significant for discrimination (see Table 1), so those VIP values are not represented. Cholesterol esters (CE); Diacylglycerols (DAG); Free Fatty Acids + Cholesterol (FFA + CHOL); Glucosylceramide (GlucCer); Long-chain FA (LCFA); Medium-chain FA (MCFA); Monoacylglycerols (MAG); Monounsaturated fatty acids (MUFA); Phosphatidic acid (PA); Phosphatidylcholine (PC); Phosphatidylethanolamine (PE), Phosphatidylinositol (PI); Phosphatidylserine (PS); Polyunsaturated fatty acids (PUFA); Saturated fatty acid (SFA); Short-chain FA (SCFA); Sphingomyelin (SM); Triacylglycerols (TAG).

**Figure 2:**
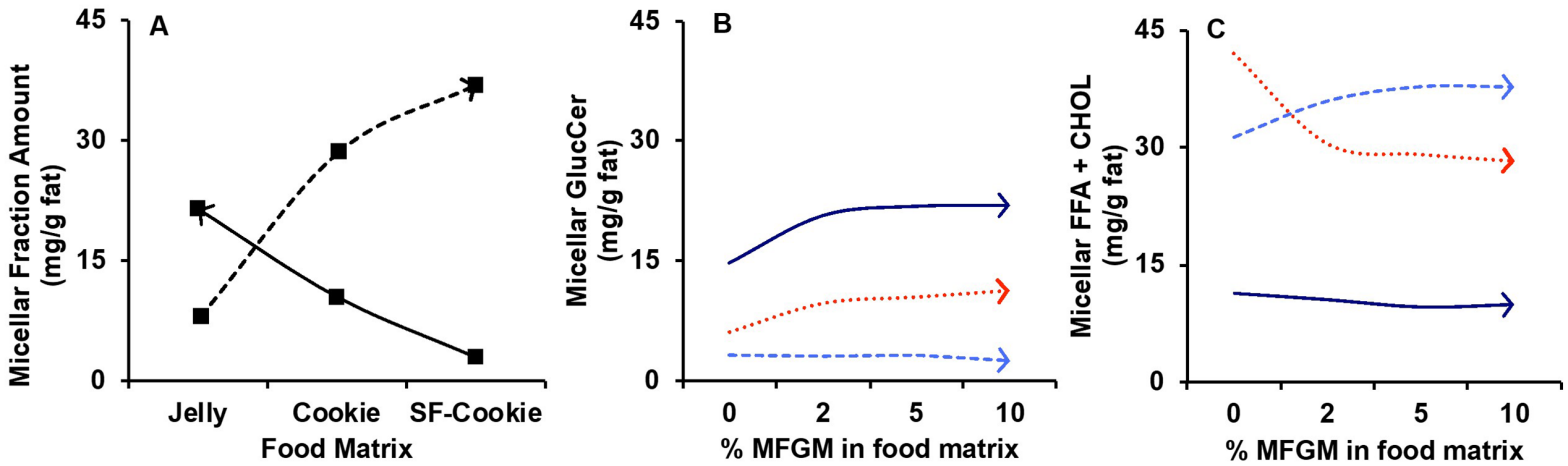
Trends observed for the influence of factors ‘Food matrix’ and ‘Supplementation level’ in lipid profile of micellar fractions obtained after digestion of MFGM-supplemented jelly, cookie, or SF-cookie. **(A)** effect of the ‘Food matrix’ factor in glucosylceramides (GlucCer, continuous line) and free fatty acids plus cholesterol (FFA + CHOL, dashed line). The effect observed in **(A)** is reinforced by the ‘Supplementation level’ for GlucCer **(B)** and FFA + CHOL **(C)** considering jelly (dark blue continuous line) and SF-cookie (light blue dashed line).

For additional understanding, we introduced the ‘Supplementation level’ (%) as a second factor to comprehend its influence on the lipid profile of the micelles after digestion, as well as the potential interaction effect with the ‘Food matrix’ factor. For the micellar lipid profiles, the evaluation of the effects of both factors was made with multivariate analysis, in which results are represented in Table 3. ‘Food matrix’ and ‘Supplementation level’ factors had distinctive effects on different lipid features using the *p*-value after adjustment by false discovery rate (FDR) approach. These data helped unravel whether common patterns for the evolution of lipid profiles exist or not. Considering the influence of both factors and their interaction as detailed in Table 3, two trends were denoted in the data (Figure 2). The first trend was that the ‘Food matrix’ factor positively or negatively affected the amount of lipids in the micellar fractions. This effect is depicted in Figure 2A for GlucCer and FFA + CHOL. Jelly micelles showed a positive trend for GlucCer and negative trend for FFA + CHOL, whereas SF-cookie micelles showed a negative trend for GlucCer and positive trend for FFA + CHOL. The ‘Supplementation level’ factor reinforced the positive trends in the association between a specific food matrix and lipid class (Figures 2B,C, for GlucCer and FFA + CHOL, respectively). Therefore, the ‘Food matrix’ × ‘Supplementation level’ interaction is positive in these cases. However, when the trend of the ‘Food matrix’ in a particular lipid feature is negative, the ‘Supplementation level’ effect does not produce any interaction (see GlucCer micellarization in SF-cookie food matrix, Figure 2B, and FFA + CHOL micellarization in jelly food matrix, Figure 2C).

**Table 3:** ANOVA fixed effects statistics of lipids determined in micelles and in cell cultures, considering differences between the factors “Food matrix”, “Supplementation level” (%), or their interaction.

	Food matrix		Supplementation %		Food matrix × Supplementation %	
	raw-P^a^	FDR^b^	raw-P	FDR	raw-P	FDR
Lipids in micelles						
CE	<0.01	<0.01	<0.01	<0.01	<0.01	<0.01
TAG	<0.01	<0.01	0.18	0.35	0.30	0.38
DAG	<0.01	<0.01	<0.01	0.05	<0.05	<0.05
FFA + CHOL	<0.01	<0.01	0.42	0.52	0.46	0.53
MAG	<0.01	<0.01	0.35	0.50	0.56	0.61
GlucCer	<0.01	<0.01	<0.01	<0.05	<0.01	<0.01
PA	<0.01	<0.01	0.08	0.30	<0.01	<0.05
PC	0.12	0.12	<0.01	<0.05	0.75	0.77
SM	<0.01	<0.01	0.10	0.31	<0.05	0.06
Ceramides	<0.01	<0.01	0.24	0.37	<0.01	<0.01
Σ PL	<0.01	<0.01	<0.05	0.07	0.06	0.12
CHOL	<0.01	<0.01	0.23	0.37	0.22	0.29
Σ Polar lipids	<0.01	<0.01	0.12	0.31	<0.01	<0.05
Σ Neutral lipids	<0.01	<0.01	0.70	0.74	0.21	0.29
C4:0	<0.01	<0.01	0.85	0.85	0.81	0.81
C5:0	<0.01	<0.01	0.16	0.31	0.57	0.61
C10:0	<0.05	<0.05	0.70	0.74	0.10	0.17
C14:0	<0.05	<0.05	<0.05	0.09	0.12	0.18
C16:0	<0.05	<0.05	0.40	0.52	<0.01	<0.05
C18:0	<0.05	<0.05	0.49	0.59	<0.01	<0.05
C16:1c9	<0.01	<0.05	0.72	0.74	0.07	0.12
C18:1c9	<0.01	<0.01	0.15	0.31	<0.01	<0.05
C18:2 c9c12	<0.01	<0.01	0.23	0.37	<0.01	<0.05
SFA	<0.01	<0.01	0.25	0.37	<0.01	<0.05
MUFA	<0.01	<0.01	0.15	0.31	<0.01	<0.05
PUFA	<0.01	<0.01	0.15	0.31	<0.01	<0.05
SCFA	<0.01	<0.01	0.05	0.22	0.22	0.29
MCFA + LCFA	<0.01	<0.01	0.12	0.31	<0.01	<0.05
Lipids in cell culture						
C4:0	<0.01	<0.05	0.46	0.76	<0.05	0.14
C5:0	<0.01	<0.01	0.13	0.48	0.05	0.18
C10:0	<0.01	<0.01	0.51	0.76	0.06	0.19
Palmitic acid	<0.01	<0.01	0.26	0.76	0.15	0.37
Palmitoleic acid	<0.01	<0.05	0.05	0.32	0.22	0.47
Stearic acid	<0.01	<0.01	0.59	0.76	0.17	0.38
C18:1 c9	<0.01	<0.01	0.15	0.50	0.11	0.30
SFA	<0.01	<0.01	0.55	0.76	<0.01	<0.05
MUFA	<0.01	<0.01	<0.05	0.32	<0.01	<0.01
PUFA	<0.01	<0.01	<0.01	<0.01	<0.01	<0.05
SCFA	<0.01	<0.01	0.80	0.83	<0.05	0.06
MCFA+LCFA	<0.01	<0.01	0.09	0.39	<0.01	<0.01

a raw-P value according to ANOVA.

b *p*-value adjusted by false discovery rate.

### 3.2. Cell cultures

**Figure 3:**
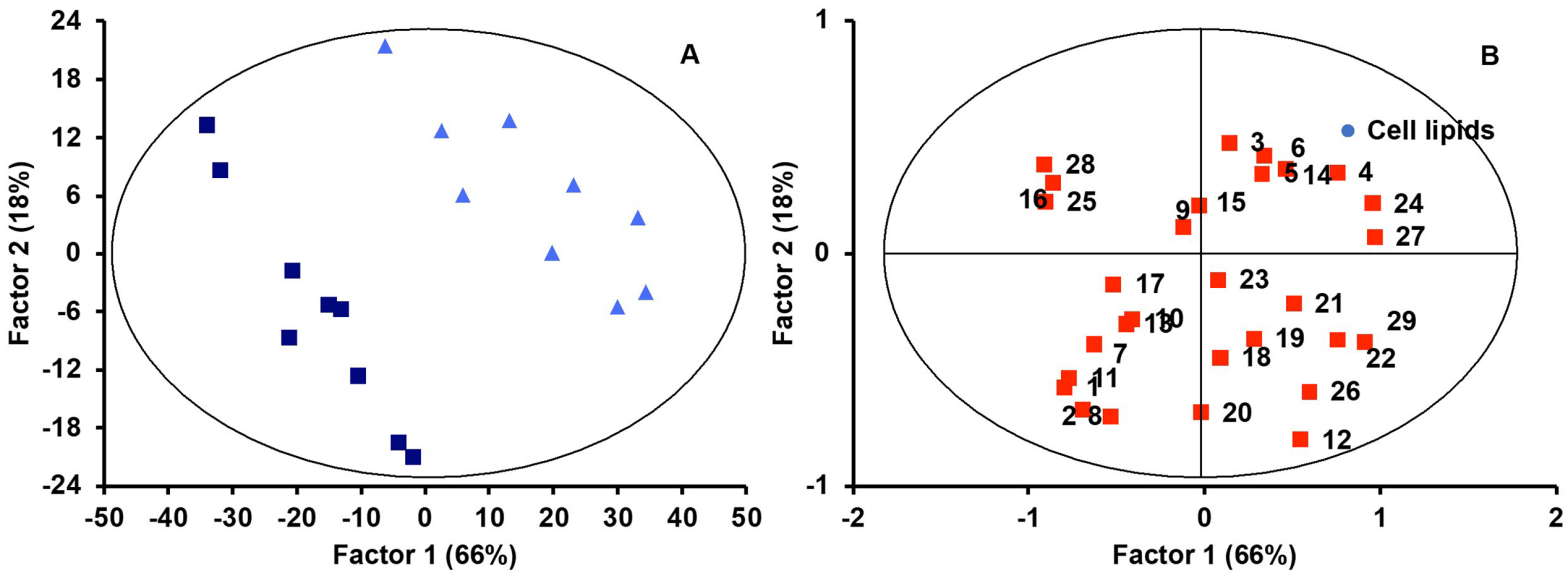
PLS-DA scores **(A)** and correlation loadings **(B)** for lipid profiles in cell culture considering the ‘Micellar profile’ factor (micelles from digested jelly, dark blue squares; micelles from digested SF-cookie, light blue tringles). Lipid features are identified with numbers as follows: 1, Cholesterol esters (CE); 2, TAG; 3, DAG; 4, FFA + CHOL; 5, MAG; 6, Glucosylceramides (GlucCer); 7, PA; 8, PC; 9, SM; 10, ƩPL; 11, Ceramides; 12, ƩNeutral lipids; 13, ƩPolar lipids; 14, CHOL; 15, C4:0; 16, C5:0; 17, C10:0; 18, C14:0; 19, C16:0; 20, C16:1 c9; 21, C18:0; 22, C18:1 c9; 23, C18:1 c11; 24, C18:2 c9 c12; 25, SFA; 26, MUFA; 27, PUFA; 28, SCFA; 29, MCFA + LCFA.

**Figure 4:**
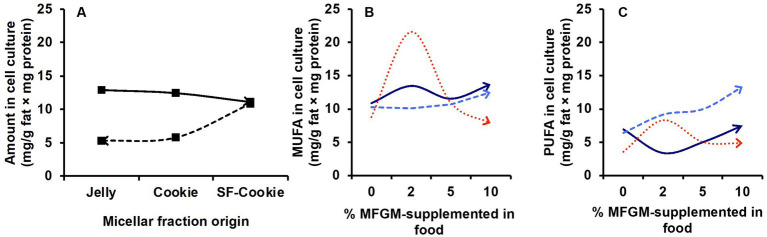
Trends observed for the influence of factors ‘Micellar fraction’ and ‘Supplementation level’ in lipid profile of cell cultures incubated with micellar fractions obtained after digestion of MFGM-supplemented jelly, cookie, or SF-cookie. **(A)** Effect of the ‘Food matrix’ factor in MUFA (continuous line) and PUFA (dashed line). The effect observed in **(A)** is reinforced by the ‘Supplementation level’ for MUFA **(B)** and PUFA **(C)** considering jelly (dark blue continuous line) and SF-cookie (light blue dashed line), while cookie (red dotted lines) showed an irregular behavior.

An interesting aspect of this study is the monitoring of the same lipid features that were previously analyzed in the micellar fraction in intestinal cell culture to observe correlations between lipid profiles in micelles and those of the cell culture after incubation. The first objective here was to determine whether the lipid profiles of the cell cultures were a predictable variable based on the type of food matrix micelles with which the cells were incubated. Applying the same statistical analysis as detailed before, a PLS-DA model was built for lipid features in Caco-2 cells. We observed that the origin of the micellar fraction could still be ascertained when comparing cell cultures incubated with micelles from the digested jelly formulation and those incubated with micelles from digested SF-cookie formulation. However, cell cultures that absorbed lipids from jelly were indistinguishable from cookie cultures and cell cultures that absorbed lipids from cookies and SF-cookies were also indistinguishable (Supplementary Figure S1). The performance of the PLS-DA model is represented in Table 1. Lipid classes with significant VIP scores are represented in Table 2 for comparisons between cell cultures incubated with digested jelly and digested SF-cookie micelles. The corresponding PLS-DA model is represented in Figure 3A, while Figure 3B depicts the correlation loadings of that model. Some lipid classes with significant VIP scores correlated well with cells incubated with micelles from digested SF-cookie, including unsaturated FA, FFA + CHOL, and total neutral lipids. In contrast, saturated fatty acids (SFA), cholesterol esters (CE), and ceramides tend to be correlated with cells incubated with micelles from digested jelly. To visualize the effect of the ‘Supplementation level’ factor and the potential interaction effects with ‘Food matrix’ (considering this factor as the origin of the micelles the cells were incubated with), multivariate analysis was performed and results are represented in Table 3. ‘Food matrix’ and ‘Supplemented level’ factors had differing effects on different lipid features considering the *p*-value after adjustment by false discovery rate approach. Considering the influence of both factors and their interaction (Table 3), MUFA and PUFA were significant in cell cultures. In fact, two different trends were denoted in the data for PUFA and MUFA, both represented in Figure 4. The first one was that ‘Food matrix’ factor positively or negatively affected the amount of PUFA and MUFA in the cell culture (Figure 4A). Specifically, a positive trend for PUFA and negative trend for MUFA in cell cultures was noted across food matrix origin. The ‘Supplementation level’ factor countered the negative trend and scarcely improved the positive one when the micelles originated from digested jelly or SF-cookie; while the cells incubated with micelles from digested cookie showed an irregular trend when ‘Supplementation level’ factor was analyzed (Figures 4B,C, for MUFA and PUFA).

**Figure 5:**
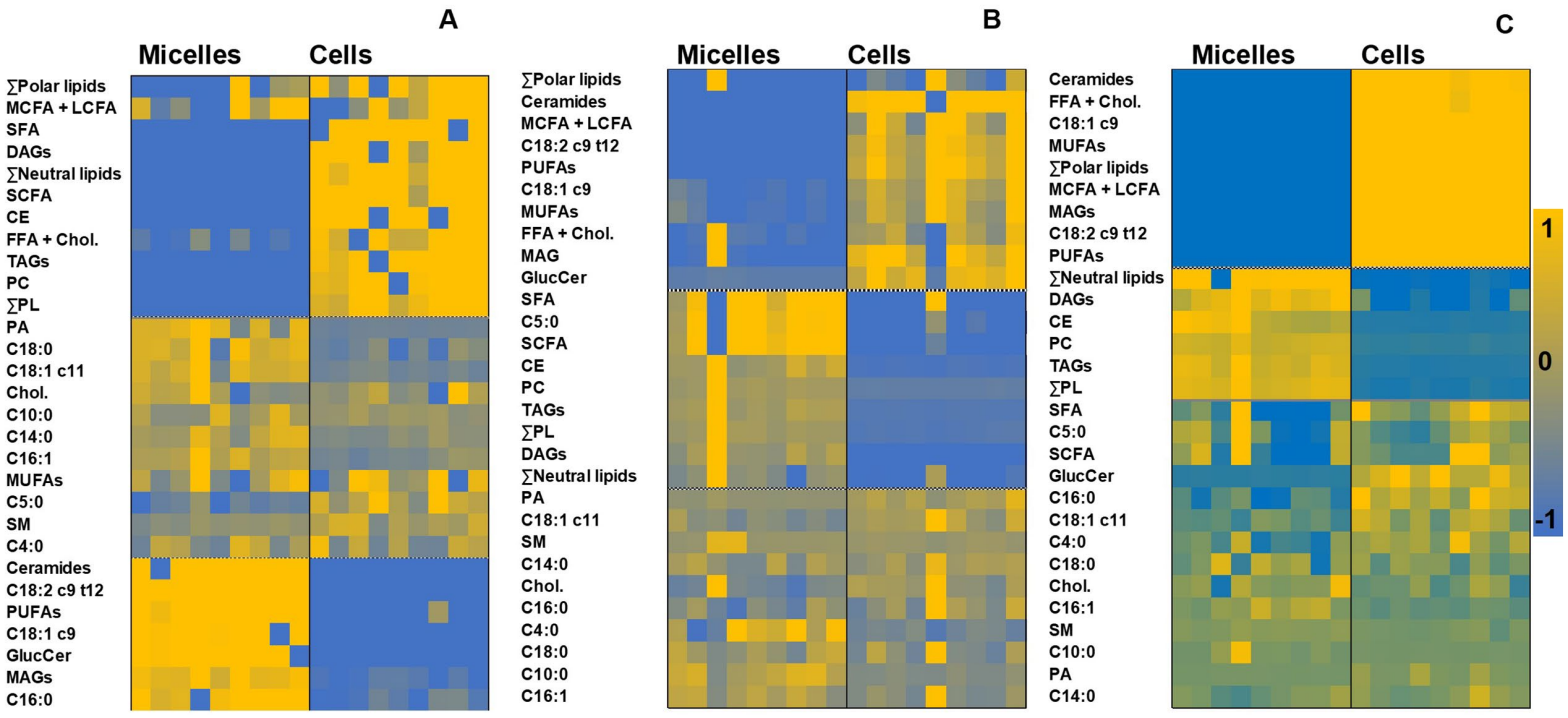
Heatmaps generated through hierarchical cluster analysis for paired comparisons of lipid profiles (normalized data) in micelles and cell cultures corresponding to digested jelly **(A)**, digested cookie **(B)**, and digested SF-cookie **(C)**. Abbreviations are the same as detailed in Figure 1.

Our subsequent objective was to monitor whether the lipid profile of the cells mirrored that of micelles from digested food matrices, or whether there were subsequent rearrangements once the lipids from micellar fractions were absorbed. A closer detail of this comparison was achieved by generating heatmaps, which group and visualize samples according to intrinsic similarities in their measurements (Figure 5). Three clusters could be observed in the heatmaps: (i) the lipid classes that increased in the cell cultures relative to their micellar fraction counterparts, (ii) those that decreased, and (iii) those remained relatively constant in both micelles and intestinal cells. Indeed, certain lipids in the micellar fraction (polar lipids, medium-chain, and long-chain FA (MCFA + LCFA), FFA + CHOL) were increased in the cell cultures, independently of the source of originating micelles. Micelles from digested jelly increased the content of SFA and neutral lipid classes, including TAG and CE in cell cultures, while the total amount of MUFA, PUFA, MAG, and ceramides increased in cell cultures after incubation with micelles from both cookie formulations.

## 4. Discussion

These findings demonstrate that food matrix features and formulations have a discriminant impact on the lipids that are micellarized for assimilation. Stemming from the complex lipid profile of the MFGM, diverse physicochemical features and enzyme requirements for digestion, and the clear-cut matrix features of the formulations used in this study, the differentiation of digested MFGM supplemented foods (jelly, cookie, or SF-cookie) was observed, despite MFGM composition remaining the same.

Food matrix effects on digestibility of food components have been reported with emphasis on those food formulations where the aim is to improve the digestibility of a particular compound. This aim is the case for phytochemicals (carotenoids, chlorophylls, anthocyanins, flavonoids), vitamins, and many others (22–25). These studies limit the effect of the food matrix on digestibility of compounds with a narrow range of structural diversity, so that the correlation between digestibility and food matrix effect is not biased by complex structural factors. However, the lipid profile of MFGM is a multi-faceted composite of compounds, in a complex trilayer membrane formed by polar lipids, neutral lipids, and glycoproteins, which makes manipulating the food matrix to control digestibility of the entire lipid profile a complex challenge (26). Food matrix represented a discriminant factor improving the digestibility of some lipids and negatively affecting that of others. This phenomenon, evident through preferred incorporation of polar lipids into jelly micelles, is of particular importance as MFGM polar lipids are increasingly recognized as key contributors to the perceived health benefits of the MFGM (27). MFGM polar lipids increase myelination of neurons (2, 28), support bone density and remodeling (29), strengthen the gut mucosal barrier (30), and more. Although the ‘Supplementation level’ factor played some significant roles, ‘Food matrix’ is the key factor influencing MFGM digestibility. However, digestibility is not the end of the bioaccessibility process but imprints the metabolism of micellarized lipids in the cell cultures.

After transport across the apical membrane of enterocytes, in part through a lipid-class dependent manner, postprandial lipids will undergo one of four main metabolic processes. They can be (1) used as substrates to *de novo* synthesize TAG that are packaged into chylomicrons and secreted into the lymph, (2) stored in cytoplasmic lipid droplets, (3) metabolized for energy in the mitochondria, or (4) used as signaling molecules for activation of nuclear receptors that modulate enterocyte lipid metabolism (31). Accordingly, most postprandial FA and MAG are re-esterified into TAG in the endoplasmic reticulum for subsequent secretion, while other complex lipid classes such as CHOL and PL can be used for the transient formation of cytosolic lipid droplets, chylomicron assembly and secretion, or membrane structures. However, in this study, there was no direct correlation between the micellar lipid profiles and cell cultures after the postprandial period, suggesting the role of the cells’ metabolic machinery to regulate the fate of the digested lipids. Lipid profiles of Caco-2 enterocytes yielded comparable, but different lipid profiles from the micelles from digested jelly and cookie formulations. Micellar and cellular lipid profiles also appear different in the case of the SF-cookie formulation.

The dynamic response of the intestinal epithelium to dietary lipids has been studied regarding the inflammatory response (32), release of hormones and their functional effects (33), and the activation of transcription factors and subsequent gene expression (34). Dietary lipids serve as signaling molecules and potent ligands for transcription factors, such as peroxisome proliferator-activated receptors (PPARs) and liver X receptors (LXR), that act as sensors to nutritional stimuli. For example, PL and specific PUFA are ligands for PPARα activation, which leads to a downstream reduction in chylomicron secretion thus reducing postprandial plasma lipid levels (35, 36). Conversely, CHOL derivatives and PUFA resulting from lipid digestion activate LXRα, which boosts TAG synthesis in enterocytes and chylomicron secretion to peripheral tissues (37). However, it is the metabolic flux between pathways that controls the ultimate fate of dietary-derived lipids. The present study suggests the involvement of the micellar lipids shifting the metabolism of the enterocytes. Specifically, we reveal that different micellar lipid profiles result in rearrangements of the cellular lipid content different from the respective micelles’ lipid profile. That is, cells sense dietary lipids and respond according to the composition of that profile. In the case of lipid micelles dominated by polar lipid classes, which are related to MFGM supplementation, it seems evident that enterocytes sense and accumulate them along with neutral lipids (i.e., TAG, CE) that may be involved in cytosolic lipid droplet formation. Relatedly, it has been demonstrated that cytoplasmic lipid droplet assembly and size is dependent on the availability of polar lipids for packaging and emulsification (38). However, if the micellar lipid profile is dominated by products derived from enzymatic hydrolysis of TAG, then intestinal cells sense these constituents and trigger the accumulation of building blocks for TAG synthesis. This phenomenon was observed for the SF-cookie food matrix rich in TAG where enterocytes accumulated MUFA, PUFA, and MAG, despite being enriched with the same MFGM and in the same percentage levels as food matrices lower in fat content. Although enriched in TAG building blocks, the absence of increased TAG accumulation in these cells may suggest the formation and secretion of lipoproteins. However, this process is directly influenced by the degree of Caco-2 polarization and differentiation (39), which may be limited as these cells were grown on standard culture plates rather than Transwell inserts.

Although the aim of the study was focused in the lipid fraction of the MFGM, it should be noted that bioactive peptides, released from hydrolytic reactions of the MFGM proteins, and MFGM proteins recalcitrant to gastric digestion (lactadherin and mucin), also might contribute to the metabolic cellular response and enhance other stimuli, including antibacterial and anti-inflammatory activities (40). Recently, bioactive peptide sequences have been identified after *in vitro* digestion of cheese and their potential bioaccessibility measured as well (41). Therefore, a complete approach to the nutritional importance and metabolic impact of the MFGM in Caco-2 cells should include fate of the MFGM proteins during digestion.

## 5. Conclusion

In recent years, numerous health benefits derived from MFGM highlight its importance as a key ingredient for food supplementation. In this study, we focused on how different food matrices (jelly, cookie, and SF-cookie) and different levels of MFGM supplementation can affect the bioaccessibility of MFGM lipids, along with their assimilation using Caco-2 cells as an intestinal epithelium model. Regarding the bioaccessibility of MFGM, characteristics and formulations of the food matrix have a greater impact on the micellarization of MFGM lipids for their assimilation than the level of supplementation provided. In this way, three differentiated micellar lipid profiles were determined according to their respective food matrix. Likewise, these micellar lipid profiles differed from cellular lipid profiles after the postprandial period, indicating that cells are capable of sensing and adjusting their lipid content based on the lipid profile of the micelles. The lipid micelles with an abundance of polar lipid classes (related to MFGM supplementation) produced an increase in neutral lipids in the cells that might be related to the formation of cytosolic lipid droplets. If the micelles are enriched in products derived from the enzymatic hydrolysis of TAG, enterocytes trigger the accumulation of basic components for the synthesis of the TAG.

It is worth noting the ability of enterocytes to sense and reorganize the lipid metabolism based on the micellar lipid profile provided. This aspect is worthy of consideration when determining which formulation is best suited to use with MFGM as a food supplement. This should be accomplished with biomolecular tools to verify the transcription factors that are up/down regulated and establish a causal relationship between micellar lipid profile and cell sensing. Additionally, the analysis of the *in vitro* digestion of MFGM directly would offer knowledge regarding the mechanisms for bioactivity, considering the complete composition (lipids and proteins) that the MFGM comprises.

## Data availability statement

The raw data supporting the conclusions of this article will be made available by the authors, without undue reservation.

## Author contributions

AP-G, JF, and RJ-F contributed to conception and design of the study. AP-G, EK, and VM-S wrote the first draft of the sections of the manuscript. EK, VM-S, and MC performed the experiments and did the analyses. MC, JF, and AP-G organized the lipid dataset and AP-G made the statistical analysis. All authors contributed to manuscript revision, read, and approved the submitted version, ensuring that issues related to accuracy or integrity of the work are adequately resolved.

## Funding

This research is part of the project PID2020-114821RB-I00, funded by MCIN/AEI/10.13039/501100011033 and by “ERDF A way of making Europe.” This project was also supported by the J.T. “Stubby” Parker Endowment in Dairy Foods at The Ohio State University (Columbus, OH; grant number 0100).

## Conflict of interest

The authors declare that the research was conducted in the absence of any commercial or financial relationships that could be construed as a potential conflict of interest.

## Publisher’s note

All claims expressed in this article are solely those of the authors and do not necessarily represent those of their affiliated organizations, or those of the publisher, the editors and the reviewers. Any product that may be evaluated in this article, or claim that may be made by its manufacturer, is not guaranteed or endorsed by the publisher.
