# Supplementary material for: Food matrix impacts bioaccessibility and assimilation of acid whey-derived milk fat globule membrane lipids in Caco-2 cells
Source: Front Nutr. 2023 May 9;10:1177152. doi: 10.3389/fnut.2023.1177152 (PMC10203207; doi:10.3389/fnut.2023.1177152)
Supplement: Supplementary file 1 [file Data_Sheet_1.docx]

Supplementary Material

Food matrix impacts bioaccessibility and assimilation of acid whey-derived milk fat globule membrane lipids in Caco-2 cells

Pérez-Gálvez, A.*, Kosmerl, E., Martínez-Sánchez, V., Calvo, M.V., Jiménez-Flores, R., Fontecha, J.

* Correspondence:

Antonio Pérez-Gálvez
aperez@ig.csic.es

# Supplementary Table

| **Table S1.** Lipid composition of AW-MFGM ingredient. | | |
| --- | --- | --- |
| Total Lipids (%) | | 6.17 ± 2.02 |
| Neutral Lipids (% of Total Lipids) | | 74.63 ± 0.78 |
|  | TAGs | 48.91 ± 1.68 |
|  | DAGs | 14.25 ± 1.27 |
|  | MAGs | 0.28 ± 0.07 |
|  | FFA + Chol. | 9.34 ± 1.37 |
|  | CE | 0.32 ± 0.13 |
| Polar Lipids (% of Total Lipids) | | 25.37 ± 0.78 |
|  | PC | 6.88 ± 0.01 |
|  | PE | 11.42 ± 0.28 |
|  | PI | 1.26 ± 0.03 |
|  | PS | 1.00 ± 0.14 |
|  | SM | 4.82 ± 0.66 |
|  | GluCer. | 0.71 ± 0.06 |
|  | LacCer. | 0.83 ± 0.05 |
| Fatty Acids (% of Total Lipids) | |  |
|  | Σ SFA | 68.22 ± 4.25 |
|  | Σ MUFAs | 26.43 ± 2.90 |
|  | Σ PUFAs | 5.36 ± 1.34 |
|  | C4:0 | 1.49 ± 0.18 |
|  | C6:0 | 1.67 ± 0.86 |
|  | C8:0 | 0.99 ± 0.39 |
|  | C10:0 | 2.32 ± 0.82 |
|  | C12:0 | 3.39 ± 0.90 |
|  | C14:0 | 9.56 ± 0.59 |
|  | C16:0 | 34.56 ± 1.09 |
|  | C18:0 | 11.88 ± 0.28 |
|  | C18:1 c9 | 21.05 ± 0.57 |
|  | C18:2 c9c12 | 5.36 ± 1.34 |
| Abbreviations are the same as detailed in Table 2. | | |

# Supplementary Figure


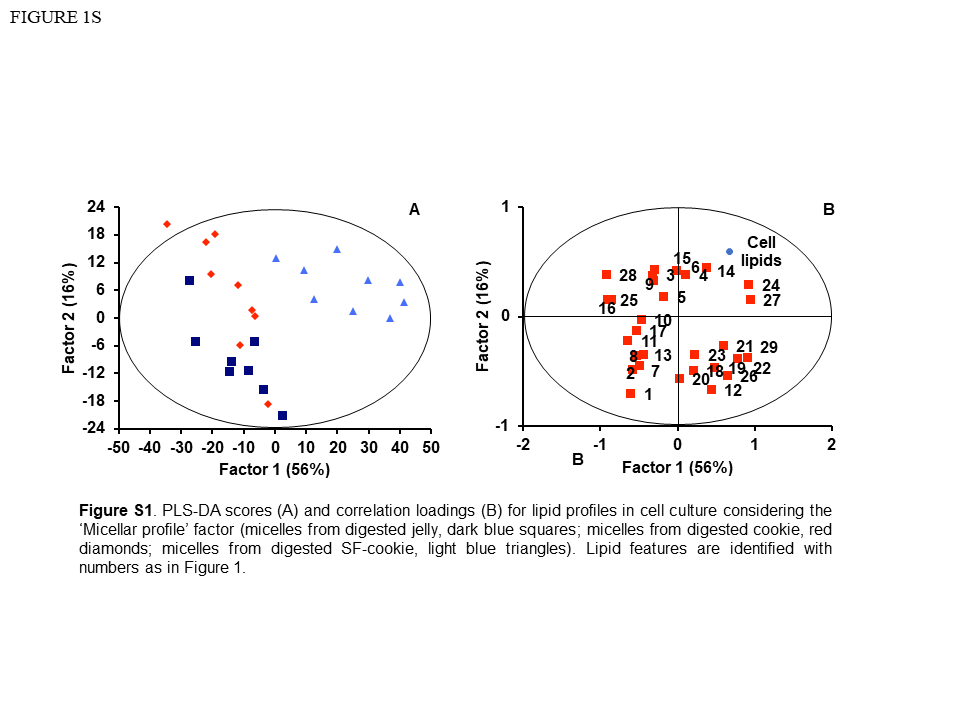


**Figure S1.** PLS-DA scores (A) and correlation loadings (B) for lipid profiles in cell culture considering the ‘Micellar profile’ factor (micelles from digested jelly, blue squares; micelles from digested cookie, green diamonds; micelles from digested SF-cookie, red triangles). Lipid features are identified with numbers as in Figure 1.
